# *In Vivo* Study on Site of Action of Sinapine Thiocyanate following Acupoint Herbal Patching

**DOI:** 10.1155/2018/9502902

**Published:** 2018-03-14

**Authors:** Shan Chen, Yu-Tong Jin, Zheng-Yang Zhu, Ling-Tao Wu, Ping Yang, Ping Jin, Li-Hua Xuan

**Affiliations:** ^1^Department of Acupuncture and Moxibustion, The First Affiliated Hospital of Zhejiang Chinese Medical University, No. 54 Youdian Road, Hangzhou, Zhejiang 310006, China; ^2^The First School of Clinical Medicine of Zhejiang Chinese Medical University, No. 548 Binwen Road, Hangzhou, Zhejiang 310053, China; ^3^Hangzhou Dianzi University, No. 1158 Second Avenue, Xiasha District, Hangzhou, Zhejiang 310018, China; ^4^The Third Affiliated Hospital of Zhejiang Chinese Medical University, No. 219 Moganshan Road, Hangzhou, Zhejiang 310005, China

## Abstract

**Objective:**

To investigate the site of action of sinapine thiocyanate (ST), following acupoint herbal patching (AHP).

**Methods:**

Twenty Wistar rats were randomized into five groups (groups A, B, C, D, and E), and all groups received the same AHP* in vivo*. Skin samples were excised at 2 h, 4 h, 6 h, 10 h, and 26 h after AHP administration from group A to group E separately and the concentrations of ST in the skin were determined using a liquid chromatography-mass spectrometry/mass spectrometry (LC-MS/MS) method. A pharmacokinetic profile of ST following AHP was performed at the same time in a group of five Wistar rats to detect plasma levels at the same time intervals.

**Results:**

The mean ± SD ST concentrations (ng/ml) at 2 h (group A), 4 h (group B), 6 h (group C), 10 h (group D), and 26 h (group E) after AHP administration were 250.01 ± 61.99, 61.01 ± 30.41, 40.12 ± 26.94, 78.66 ± 59.43, and 19.55 ± 18.95, respectively. No ST was detected in rats' plasma samples at the same time points.

**Conclusions:**

The site of action of ST following AHP is in the skin.

## 1. Introduction 

Acupoint herbal patching (AHP), also known as “xueweitiefu” in Chinese, is an external therapy which requires the application of Chinese herbal patches to certain acupuncture points on the body [1]. This therapy has been used in China for thousands of years to prevent and treat diseases [2]. The use of AHP was first recorded in* the Prescriptions for Fifty-Two Diseases (Wu Shi Er Bing Fang)*, written in approximately the fourth century BC and considered the oldest herbal text in traditional Chinese medicine (TCM) [3]. The prescription of AHP to treat asthma was originally recorded in* Zhang Shi Yi Tong*, a medical book written by Zhang Lu from the Qing Dynasty [4]. Currently, in China, it is mainly used to treat respiratory diseases such as asthma and allergic rhinitis for its satisfactory effects [5].

It is believed that AHP can stimulate the skin at specific acupuncture points and the qi of zang-fu organs and meridians (pathways in which the qi and blood of the human body are circulated). The application of herbal patches may regulate the functional activities of body and in turn prevent and treat diseases based on the theory of TCM [6]. Modern research has been conducted to investigate the mechanisms of AHP for asthma and allergic rhinitis. A review and meta-analysis showed that AHP improved the forced expiratory volume in 1 second (FEV1) in asthma patients [7]. Another review including six studies suggested that AHP showed favorable immunomodulatory effects for the treatment of childhood asthma [8]. Rat experiments in asthma have indicated that the mechanism of AHP may be related to regulating the expression levels of transcription factors [9]. However, it is unknown whether the components of herbal patches are retained in the skin or penetrate across the skin and absorbed into the blood circulation following AHP. White mustard/Bai Jie Zi* (Sinapis alba)* has a long history of medicinal use in China for cough and asthma relief and it is the primary medicine in the prescription for AHP [10].

In the current study, we selected sinapine thiocyanate (ST) as the major marker of white mustard/Bai Jie Zi* (Sinapis alba)* to investigate the site of action following AHP. We determined the concentrations of ST in the skin and plasma through* in vivo* skin permeation profile and pharmacokinetics analysis.

## 2. Materials and Methods

### 2.1. Design

This was a randomized animal trial involving Wistar rats conducted from March 2016 to July 2016.

### 2.2. Medicines and Reagents

#### 2.2.1. Preparation of Herbal Patches

The formula for herbal patches contains raw white mustard/Bai Jie Zi* (Sinapis alba)*, Yan Hu Suo* (Rhizoma Corydalis)*, Xi Xin* (Herba Asari)*, and Gan Sui* (Radix Kansui),* which were all purchased from the pharmacy at Zhejiang Provincial Hospital of TCM. More than 200 grams of the above-mentioned crude drugs were pulverized (100 mesh) at a ratio of 1 : 1 : 0.5 : 0.5, respectively, and then blended with fresh ginger juice (purchased from local hypermarket) to make herbal patches of 0.7 cm × 0.7 cm × 0.3 cm (length × width × height), with a mean ± standard deviation (SD) weight of 1 ± 0.04 g each. The content of ST in one patch was determined to be 6477.72 ug. The patches were then laid on adhesive nonwoven fabrics of 2 × 2 cm before use.

#### 2.2.2. Reagents

The standard ST was obtained from the National Institute for the Control of Pharmaceutical and Biological Products of China (batch number: 111702-201504). The internal standard ribavirin was manufactured by Sigma (Sigma-Aldrich Co. LLC., MO, USA). Methanol and acetonitrile of high performance liquid chromatography (HPLC) grade were from Merck & Co. Inc. Ultrapure water was prepared by Thermo Barnstead Ultrapure water purifier (Thermo Fisher Scientific Co., USA). All other reagents like ethanol and sodium chloride were of analytical grade.

### 2.3. Instruments

Analysis was performed using an ABI (Applied Biosystems/SCIEX, USA) 4000 Qtrap mass spectrometer equipped with a Turbo V electrospray ion (ESI) source, a triple quadrupole linear ion trap analyzer, and data processing software Analyst (Version 1.4). An ACQUITY ultraperformance liquid chromatography (UPLC) system (Waters Co., USA) was equipped with a binary pump and an autosampler.

### 2.4. Animals

Healthy Wistar rats (males, 6 weeks old, weight of 150–200 g) of a specific-pathogen-free (SPF) level were provided by the Center of Laboratory Animals, Zhejiang Chinese Medical University. Animal experiments were performed in accordance with the Principles of Laboratory Animal Care and Use in Research published by the Chinese Ministry of Health [11]. The animals were kept in well-spaced cages under well-controlled conditions (21–25°C and humidity of 60–70%); they were subjected to a 12-h day-night cycle (lights were turned on at 8 a.m.). All animals were supplied with normal pellet food and water.

### 2.5. Penetration Studies

#### 2.5.1. Groups

Twenty rats were randomly divided into five groups (*n* = 4), namely, groups A, B, C, D, and E. All five groups received the same AHP for the same duration (2 hours).

#### 2.5.2. *In Vivo* Skin Penetration

The hair on the dorsal side of each group was clipped off and a depilatory cream was used to remove the fine hair. The skin was then rinsed topically with normal saline (NS) solution and dried with gauze. When the rats were prepared, an area of 0.7 × 0.7 cm^2^ was marked on every acupuncture point, namely, Feishu (BL13), Pishu (BL20), and Shenshu (BL23), on both sides [12, 13], and AHP was applied.

After 2 hours, herbal patches were removed from each group and its residue on the dorsal side of each animal was cleared away. Group A was immediately anesthetized using an intraperitoneal (i.p.) injection of 7% chloral hydrate solution (0.5 mL/100 g). The skin samples were excised from the marked area on the acupuncture points, and the hair and subcutaneous fat were removed. The skin was rinsed with NS solution and dried with paper again before freeze preservation at −80°C. The skin samples in groups B, C, D, and E were excised following the above steps at 4 h, 6 h, 10 h, and 26 h after AHP administration, respectively. All specimens were stored at −80°C. At the end of sampling, all specimens were minced with ophthalmic operating scissors for redissolution and combined with 3 mL of 10% (v/v) ethanol in NS solution (pH 5.5) for homogenization. Thereafter, they were centrifuged for 10 minutes (3000 rpm). The supernatant was then filtered through a millipore (0.22 *μ*m) filter, and the subsequent filtrate was collected to determine the concentrations of ST in the skin using a liquid chromatography-mass spectrometry/mass spectrometry (LC-MS/MS) method.

#### 2.5.3. Pharmacokinetic Profile

Five healthy Wistar rats of a SPF level were fasted for over 12 hours but had free access to water. AHP was applied for 2 hours on the same acupoints of the rats as those in the skin penetration study. Approximately 0.3 to 0.5 mL of blood samples were collected from the orbital venous plexus using glass capillary and put in heparinized tubes before (0 min) and after the administration of AHP (2 h, 4 h, 6 h, 10 h, and 26 h). The samples were centrifuged for 10 minutes at a speed of 3600 rpm to obtain separated plasma. All samples were stored in a refrigerator (−80°C) before use. The plasma concentration of ST at each time point was determined using the LC-MS/MS method.

### 2.6. LC-MS/MS

#### 2.6.1. Chromatographic Conditions

The separation was performed on a Capcell PAK MG II C18 column (100 mm × 2.0 mm, 3 *μ*m; Shiseido Co., Ltd., Japan) with a mixed mobile phase of 0.1% formic acid in water (v/v) (A) and acetonitrile (B). The gradient elution was programmed as follows: 0.0–1.0 min, 12% B; 1.0–4.0 min, 12–60% B; 4.0–4.1 min, 60–12% B; 4.1–5 min, 12% B. The column temperature was maintained at 30°C, and the flow rate was set at 0.2 mL/min with an injection volume of 1 *μ*L.

#### 2.6.2. Mass Spectrum Conditions

The Electrospray Ionization (ESI) was operated in the positive ion mode under Multiple Reaction Monitoring (MRM). The mass spectrometric parameters were optimized as ion spray voltage, 5500 V; ion source temperature (TEM), 100°C; curtain gas (CUR) pressure, 1.38 × 10^5^ Pa; nebulizer pressure (gas 1), 3.45 × 10^5^ Pa; drying gas (gas 2) pressure, 3.79 × 10^5^ Pa. Nitrogen was used in all cases. The precursor-product ion pairs of the analytes and internal standard, declustering potential (DP), and collision energy (CE) are shown in Table 1.

### 2.7. Statistical Analysis

Data was analyzed using SPSS (Version 19.0) software. ST concentrations were expressed as mean ± SD. One-way analysis of variance (ANOVA) was used for intragroup comparison, and intergroup comparison was conducted using Student–Newman–Keuls tests. Quality control samples were assayed using a series of concentration gradients for the ST concentrations. The calibration curve was established by plotting the peak area ratio of ST to internal standard (IS) against the nominal concentrations. The equation of peak area (*Y*) versus concentrations (*X*) was determined by linear regression analysis.* P* values < 0.05 were considered statistically significant.

### 2.8. Pharmacokinetic Analysis

Pharmacokinetic parameters were calculated using a kinetic program according to the concentrations in plasma at different times.

### 2.9. Ethics

The animal protocol was approved by the Laboratory Animal Management and Welfare Ethical Review Committee of Zhejiang Chinese Medical University. As this study did not involve human subjects, informed consent was not required.

## 3. Results

*Calibration Curve and Lower Limit of Quantitation of Sinapine Thiocyanate. *The calibration curve demonstrated the acceptable accuracy of the ST assay that was within the range of 0.01–10 *μ*g/mL and had good linearity (Table 2).

### 3.1. Skin Penetration Profile of Sinapine Thiocyanate

**Figure 1:**
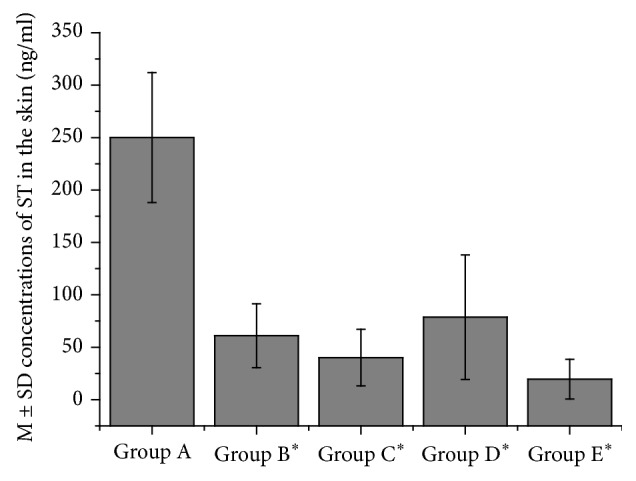
Comparison of the concentrations of sinapine thiocyanate in the skin between different time groups. Abbreviations: ST: sinapine thiocyanate; M ± SD: mean ± standard deviation. Note: ^*∗*^compared with Group A, *P* < 0.05.

The mean ± SD ST concentrations (ng/ml) at 2 h (group A), 4 h (group B), 6 h (group C), 10 h (group D), and 26 h (group E) after AHP administration from all six acupoints were 250.01 ± 61.99, 61.01 ± 30.41, 40.12 ± 26.94, 78.66 ± 59.43, and 19.55 ± 18.95, respectively (Figure 1). We found that the ST concentrations in the skin for group A were significantly higher than those for the other groups (*P* < 0.05). There were no significant differences observed between groups B, C, D, and E (*P* > 0.05).

### 3.2. Plasma Concentrations of ST

**Figure 2:**
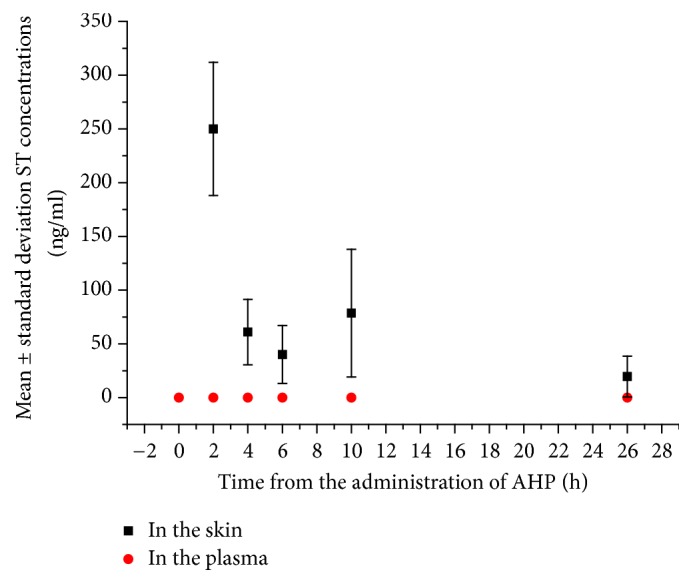
Comparison of the concentrations of sinapine thiocyanate in the skin and plasma at different time points. Abbreviations: ST: sinapine thiocyanate; AHP: acupoint herbal patching.

We did not detect ST in any of the rat plasma samples at 0 min or at 2 h, 4 h, 6 h, 10 h, and 26 h after administration of AHP. Comparisons between ST concentrations in the skin and in the plasma at different time points are shown in Figure 2.

## 4. Discussion

The AHP therapy is a type of transdermal drug delivery. To date, the mechanisms behind this therapy remain undefined; several studies have indicated that AHP took part in the immunological regulation when treating allergic rhinitis and asthma [14, 15]. With an increasing understanding of the skin as an immune system, the intradermal route has emerged as an ideal route to administer vaccines and immunomodulators [16]. Strong associations were observed between skin reactions and therapeutic efficacy by researchers [17, 18]. Outside stimuli enable skin to elicit immune responses when the physical barrier of the skin is damaged. Therefore, stimulation of the skin at specific acupoints by AHP might enables potent antigen-presenting cells (APCs), subsequently inducing immune responses in the skin [19, 20]. In light of the skin immunity theory, the investigation of the site of action of the herbal component used in AHP is fundamental. White mustard/Bai Jie Zi* (Sinapis alba)* is one of the sovereign medicines in AHP. As specified in the* Chinese Pharmacopoeia*, ST is selected as the main marker for the determination of white mustard content [21].

*In vitro *skin permeation experiments have demonstrated the permeability of ST, showing a positive correlation between time and the amount of permeation accumulated, with good linearity. It was determined that the release rate was mainly limited by the skin barrier [22]. Guo et al. discovered that after* in vivo* penetration of white mustard extract for 24 hours, ST was able to permeate across all skin layers with a maximum accumulation in the stratum corneum [23]. Xu et al. studied dermal pharmacokinetics of ST after applying a single white mustard extract using microdialysis combined with HPLC, and they found that the mean residence time was approximately 22 h and the half-life was approximately 15 h, suggesting that ST was capable of maintaining a certain concentration level [24]. To our knowledge, the determination of ST both in the skin and in the plasma using a compound prescription for herbal patches has not been done in previous studies. In the present study, the herbal patches we applied in live animals were formulated according to a complex formula. We determined the concentrations of ST in the skin and in the plasma after AHP at different time points. We observed that ST was retained in the skin following AHP administration for up to 26 h. ST was not detected in the rat plasma at the same time points after the administration of AHP.

It was also observed that the concentrations of ST in the skin changed at different time points. The concentration of ST was the greatest at 2 h after AHP (group A) and thereafter declined from groups B to E. We assume that the reduction of ST concentrations was due to the removal of AHP at 2 h and therefore no more ST could be released into the skin. However, ST was retained in the skin in groups B to E up to 24 h after AHP discontinuation and there were no significant differences (*P* > 0.05) between these groups. It was therefore determined that ST concentration levels could be maintained for at least one day after the herbal patch was withdrawn.

As the quaternary amine alkaline, ST is the principal form of sinapine in botanicals and is water-soluble [25]. Another possible reason for the decrease in the ST concentrations in groups B to E is the hydrolysis and enzymolysis of sinapine in the skin, leading to the decomposition of sinapine into sinapic acid and choline bisulfate [26]. Meanwhile, as a phenolic compound, ST may be catalyzed by polyphenol oxidase in living organisms (such as tyrosinase) to produce diphenol or quinones [27].

According to clinical practice, the AHP regimen features single treatment for 2 hours with repeated use every ten days; this process is repeated three times as one course. The ST was retained in the skin for approximately 26 h; the decline in ST concentrations over time following AHP indicates that AHP should be administered in regular intervals.

This study was subject to several limitations. Since the prescription of the herbal patch we used in our study was a complex formula, ST is only one of the components in white mustard/Bai Jie Zi* (Sinapis alba)*. The site of action for other representative constituents from Yan Hu Suo* (Rhizoma Corydalis)*, Xi Xin* (Herba Asari)*, and Gan Sui* (Radix Kansui) *was unknown. Further studies are needed to investigate the actions of the main components of these herbal medicines. We did not have a control group in order to compare skin samples from acupoints and nonacupoints. A study conducted by Liu et al. showed differences in skin stratum corneum thickness between acupoints and nonacupoints. Acupoints demonstrated different penetrating characteristics from nonacupoints [28]. However, the selection of nonacupoints remains controversial. It was reported that the range of acupoint size varies from 2.7 to 41.4 cm^2^ [29]. Because the location of nonacupoints is not unified, it is difficult to design nonacupoints as distinct from verum acupoints. Additionally, skin thickness varies in different parts of the body even without the consideration of acupoints or nonacupoints [30]. Therefore, it is necessary to elucidate these factors and design a proper control group in our future work.

## 5. Conclusions

Our results indicate that after AHP, ST can penetrate the skin and be retained beneath the acupoints application sites; ST was not detected in the plasma. As the main constituent of white mustard/Bai Jie Zi* (Sinapis alba)*, we consider that the site of action of ST following AHP occurs in the skin. The possible rationale underlying AHP may be related to the stimulation of the skin at acupoints. Further research needs to be conducted to investigate intradermal mechanisms of ST under the acupoints sites.

## Figures and Tables

**Table 1:** Retention time, ion pairs detected, and main parameters of mass spectrum.

Compounds	*t*_R_ (min)	MS_1_ (*m*/*z*)	MS_2_ (*m*/*z*)	DP (V)	CE (V)
ST	3.17	310.2	251.1	70	22
Ribavirin	1.11	267.1	135.1	197	22

*Abbreviations*. MS: mass spectrum; DP: declustering potential; CE: collision energy; ST: sinapine thiocyanate.

**Table 2:** Calibration curve and limit of quantification for sinapine thiocyanate.

Target	Linear range	Linear equation	*R* value
ST	0.01–10 *μ*g/mL	*Y* = 63.47858*X* − 170.36523	0.99770

*Abbreviations*. ST: sinapine thiocyanate; *Y*: peak areas; *X*: concentrations.
